# Active Control of the Spoof Plasmon Propagation in Time Varying and Non-reciprocal Metamaterial

**DOI:** 10.1038/s41598-018-36948-2

**Published:** 2019-02-20

**Authors:** A. Ourir, M. Fink

**Affiliations:** Institut Langevin, ESPCI Paris, CNRS, PSL University, 1 rue Jussieu, 75005 Paris, France

## Abstract

We present an efficient concept based on time varying and non reciprocal metamaterials to achieve an active control of the spoof plasmon (SP) propagation at sub-wavelength scale. An experimental demonstration of non-reciprocal guiding device based on split ring resonator is proposed as an application of this concept in the microwave regime. We show that this device is able to blue-shift the propagated SP waves and to achieve an active steering of these SPs at sub-wavelength scale by controlling the modulation frequency of the time varying metamaterial. This approach could be extended plainly to infrared and optical regimes by considering suitable technologies.

## Introduction

Surface plasmons (SPs) are electromagnetic surface waves propagating along a metal-dielectric interface that can be observed in the visible wavelength regime^1^. SPs have been proposed for tailoring the light propagation at sub-wavelength scale thanks to the strong confinement of light at the metal-dielectric interface^2^. A series of applications based on SPs have been investigated theoretically and demonstrated experimentally, such as guiding and focusing devices^3^, photonic lenses^4^, wavelength demultiplexers^5^, integrated circuits^6^ and far-field sub-wavelength imaging^7^.

Spoof plasmons have been proposed to engineer SPs at THz and microwave frequencies^8,9^. This result has been obtained by cutting grooves on a scale much smaller than the wavelength to increase the penetration of the fields into the metal. These structures have been intensively investigated and has been utilized for many wave guiding and focusing applications^10–16^. Recently, second harmonics of spoof surface plasmon polaritons have been generated efficiently at microwave frequencies using a subwavelength-scale nonlinear active device^17^. This effect has been obtained over a broad frequency band by loading the nonlinear device to the intersection of two plasmonic waveguides with different corrugation depths.

The dynamic control of metamaterial properties is attracting great interest in optics and microwaves in the last years. With the inception of time-gradient metasurfaces, it has been shown that Snells law can be modified to an even more universal form. By using this approach, new type of optical isolators has been developed by breaking Lorentz reciprocity^18^. Here, we show that the manipulation of the SP propagation can be achieved in a time varying metamaterial. This effect can be obtained in a planar subwavelength device composed of Split Ring Resonator (SRR) arrays that supports propagation of “magnetic” SPs at sub-wavelength scale. We show that a time gradient effect can be achieved by using an active resonator in the junction between three SRR based waveguides. A temporel modulation and an active manipulation of the SP propagation can be controlled by this time varying metamaterial. We show that the proposed structure is able to achieve an active switching device at a sub-wavelength scale.

## Active Control of the SSP Propagation

It has been shown recently that a time varying metamaterial induces a phase shift on transmitted waves^18^ given by:

1 $${\varphi }_{out}={\varphi }_{0}+{\varphi }_{m},$$

where *ϕ*_0_ is the phase of the incident wave and *ϕ*_*m*_ the space-time varying phase shift in the metamaterial. This leads to a time variation on the frequency of the transmitted signal given by:

2 $${f}_{out}={f}_{0}\pm \frac{1}{2\pi }\frac{\partial {\varphi }_{m}}{\partial t},$$

where *f*_0_ is the frequency of the incident wave. When we consider a normal incident wave and harmonic time variation of the metamaterial properties at a frequency *f*_*m*_, we obtain:

3 $${f}_{out}={f}_{0}\pm {f}_{m}.$$

The above equation (Eq. 3) describes the modulation that can be achieved on the input signal transported by the incident wave.

**Figure 1:**
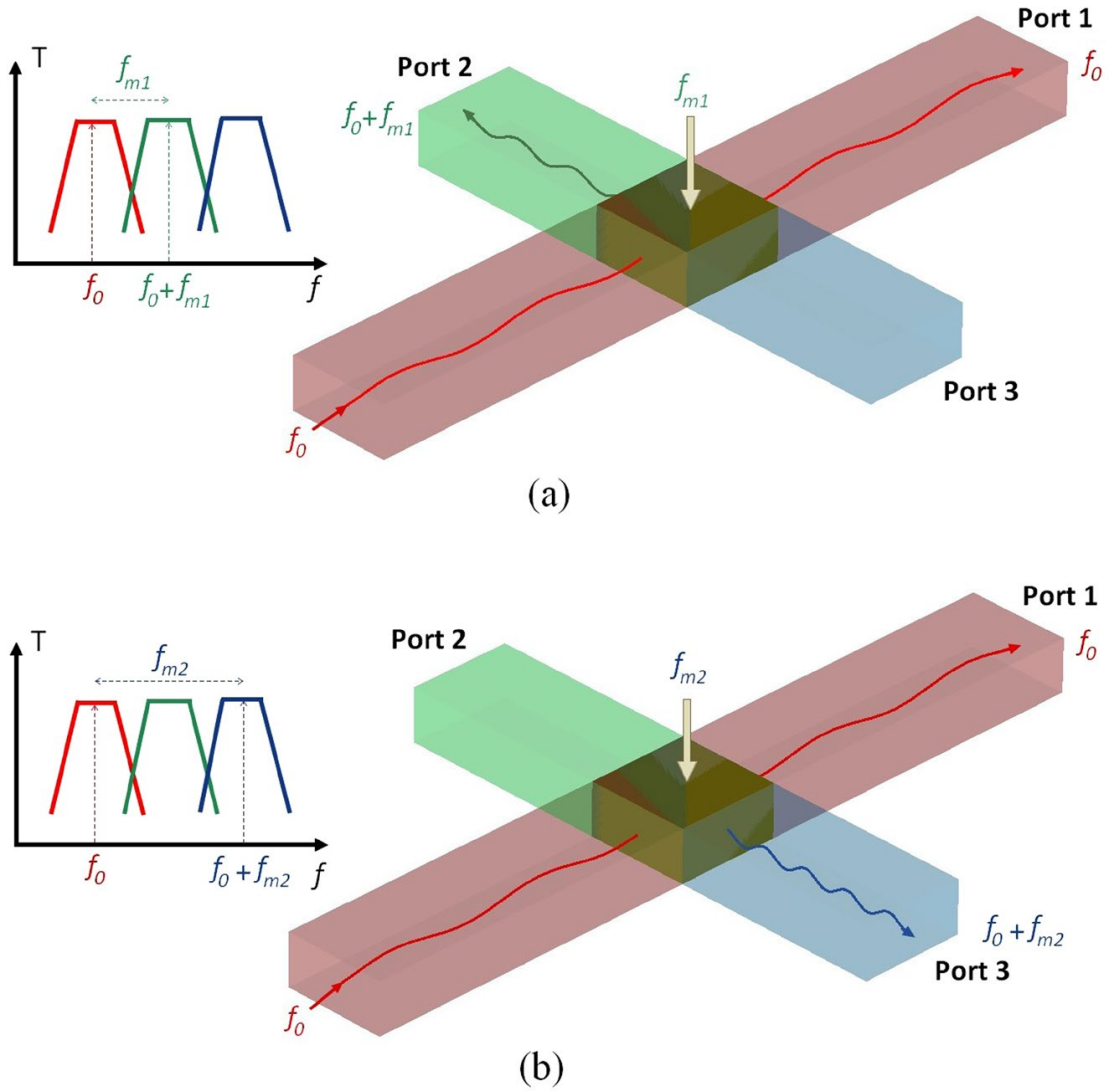
Schematic views describing the concept of the active control of the SSP propagation using a time varying junction modulated at the two frequencies *f*_*m*1_ (**a**) and *f*_*m*2_ (**b**).

Metamaterials supporting spoof plasmon wave propagation can be designed for a specific range from microwave to mid-infrared frequencies. Here, we propose to design a time varying non reciprocal subwavelength device based on such metamaterials in order to control the SP propagation. Figure 1 presents the schematic view and the functioning description of the proposed device. Three waveguides supporting SP propagation are used for the realization of the device. These meta-waveguides are bandpass filters designed to provide three different transmission frequency bands. A junction made by a time varying metamaterial is used to realize the connection between the three waveguides. This junction is dedicated to achieve the modulation of the incident SP composed of signals at the frequency *f*_0_. Depending on the value of the modulation frequency *f*_*m*_, the SP coming from the first waveguide is then directed to the second (Port 2) or the the third waveguide (Port 3). This steering effect is provided by the bandpass properties of the meta-guides. In order to reduce the reflection of the remaining signal at the frequency *f*_0_, the first waveguide is extended beyond the junction, providing a supplementary output (Port 1) and collecting such remaining signal. The time varying metamaterial junction is able to carry out a switching between the second and the third output ports by controlling the modulation frequency *f*_*m*_. Thereby an active switcher for SP could be achieved by such device.

Now that we have discussed the basic principle and the physical mechanisms behind the control of the SP propagation, we move to the next stage concerning the design of the proposed time varying non reciprocal device. SPs offers a real opportunity to realize subwavelength components for modern electronics, especially in the infrared and optical regime. A wide variety of plasmonic and metamaterial based components have been recently proposed for these regimes. We have demonstrated recently the possibility of using defect SSP modes to realize bandpass metawaveguides^19^. Such waveguides could be used here for the design of our switching device at infrared and even at optical frequencies. Electro-optical modulation could be then applied to achieve the time varying property of the metamaterial based junction. For instance, transparent conductive oxides (TCOs), specifically indium tin oxide (ITO), could be proposed to achieve this task due to their electrically-tunable permittivity in these regimes^20,21^.

## Experimental Demonstration

Here we propose to make an experimental demonstration of the SP propagation control in the microwave regime. Recently, the propagation of SP in an ultrathin metal strip consisting of connected split-ring resonators (SRRs) has been demonstrated at microwave frequencies^22^. Here, we propose to realize our subwavelength switching device by exploiting waveguides composed of SRR arrays. Such structures are very compact and can be realized conveniently by chemical, optical or electronic etching techniques^23^. Besides, grounded SRRs are able to provide painlessly bandpass filtering as it will be shown hereinafter.

One dimensional array of SRR can be modeled by using a simple electric equivalent circuit and by applying Kirchoff’s laws. Typical dispersion relation of such arrays is given by:

4 $$cos(ka)=[{(\frac{{\omega }_{0}}{\omega })}^{2}-1]/\kappa $$

where *ω*_0_ is the resonant frequency of the SRR and *κ* is the mutual coupling between SRRs^24^. With a particular SRR orientation it is possible to obtain a bandpass waveguide supporting backward wave propagation (so that the phase and group velocities have opposite signs) at sub-wavelength scale.

**Figure 2:**
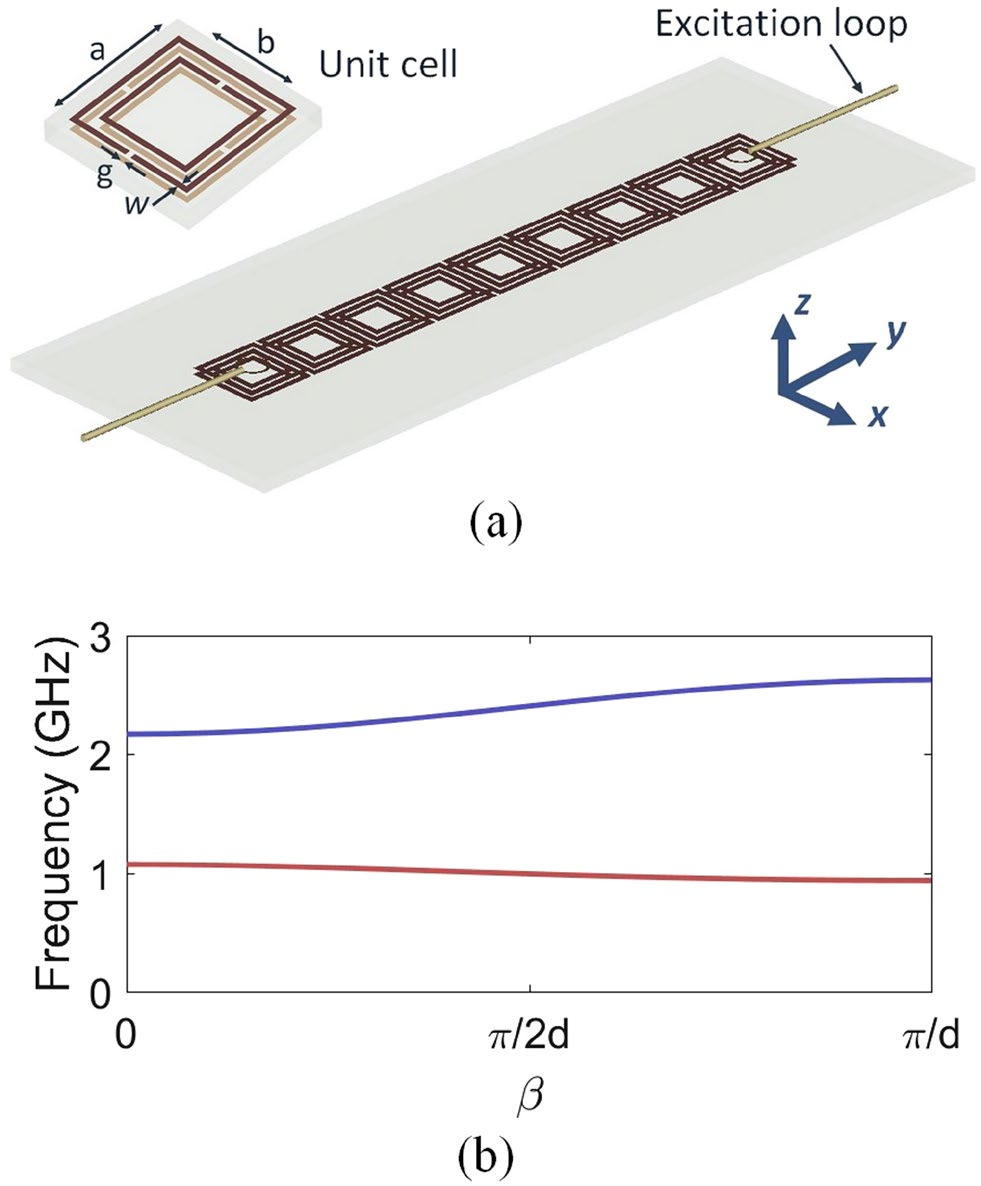
(**a**) Schematic view of the waveguide composed of a periodic array of split ring resonators. The unit cell of the array is given at the top-left side of the figure. (**b**) Calculated dispersion diagram of the structure (*a* = 13.5 mm, *b* = 11.5 mm, *w* = *g* = 1 mm). The lattice constant of the array is *d* = 14 mm.

Figure 2(a) presents a schematic view of the designed meta-waveguide. A periodic array of grounded SRRs is used for each waveguide. Two small loops are placed above the cell of each extremity of the waveguide to excite the structure. The schematic view of the unit cell is illustrated in the inset of Fig. 2(a). Four square shaped SRRs printed on a double face Epoxy substrate are considered in each cell. The array is then placed over a metallic ground plane above a 1 mm-thick Epoxy substrate.

The dispersion diagram of the proposed meta-guide has been calculated by performing Eigen-mode simulations. The two first modes are presented in Fig. 2(b). One can clearly observe the negative slope of the first mode around 1 *GHz*. This phenomenon relates the existence of the backward waves due to a negative mutual coupling as predicted by the previous dispersion relation.

**Figure 3:**
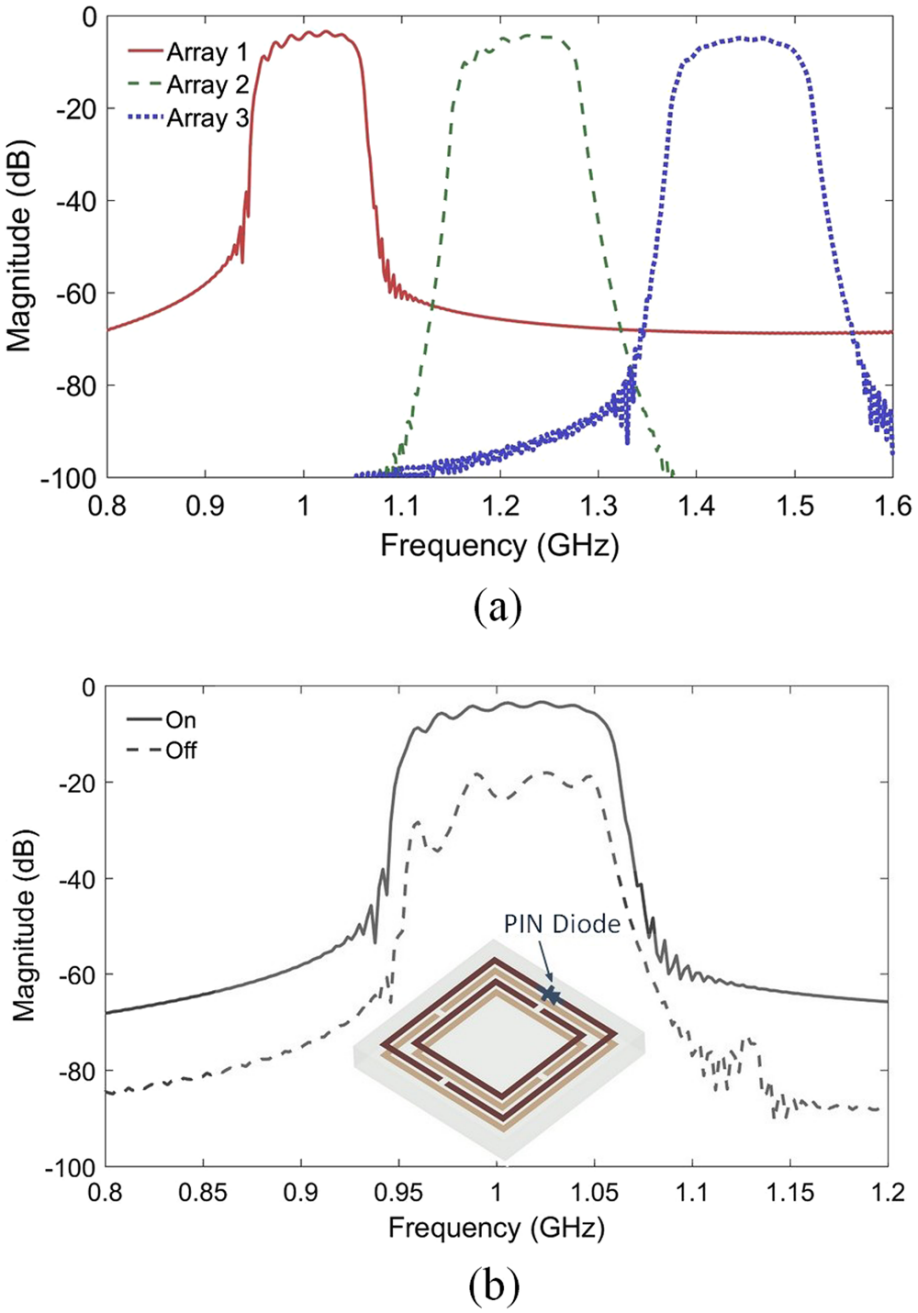
(**a**) Calculated transmission spectra of three SRR based waveguides. The lattice constant of the first array (*a* = 13.5 mm, *b* = 11.5 mm, *w* = *g* = 1 mm) is *d* = 14 mm. Different SRRs are used in the design of the second (*a* = 13 mm, *b* = 11 mm, *w* = *g* = 1 mm and *d* = 13.5 mm) and the third (*a* = 12.5 mm, *b* = 10.5 mm, *w* = *g* = 1 mm and *d* = 13 mm) arrays. (**c**) Transmission spectra of the main waveguide containing a time-varying unit cell, calculated for the two possible configurations. The inset shows a schematic view of the time-varying unit cell.

Three different arrays are then considered in order to provide three different bandpass filters for the realization of the proposed device. Time domain full wave electromagnetic simulations have been performed in order to optimize the transmission spectra of the designed meta-waveguides. Figure 3(a) shows the calculated transmission spectra for the considered SRR based waveguides. The first spectrum is related to the structure presented in Fig. 2. The obtained transmission band shown by this spectrum corresponds to the first mode of the calculated dispersion relation [Fig. 2(b)]. The three structures provide three bandpass filters operating at a frequency comprised between 1 and 1.6 GHz. These three filters are designed to direct the SP propagation at sub-wavelength scale in the final switching device.

In order to realize the time varying metamaterial, an active SRR based unit cell is designed. A PIN diode is inserted in a supplementary gap of the top printed SRR as illustrated in the inset of Fig. 3(b). In the ‘ON’ configuration of the PIN diode, the behavior of time varying unit cell is similar to each cell of the first array. While, in the ‘OFF’ position, this cell provides different behavior. In order to show the effect of such time varying cell on the transmission of meta-waveguides, we have replaced the central cell of the first meta-waveguide with this cell. We have then performed numerical calculations of the transmission spectra for this waveguide for both states of the PIN diode. Figure 3(b) shows the obtained results. An important drop of the transmission of about 15 dB is observed when switching between the two states. We have taken advantage of this phenomenon to control the time varying metamaterial. The principle is to adjust the modulation frequency *f*_*m*_ by using a sinusoidal signal at this frequency as bias voltage of the PIN diode. The switching between the two states at a frequency *f*_*m*_ should provide the time gradient effect in the proposed device.

The three meta-waveguides and the active SRR based unit cell have been realized and characterized in a semi-anechoic chamber. A spectrum analyser is used to obtain the magnitude transmission spectra of the waveguides. A software-defined radio (SDR) card is used to provide the bias voltage operating at the modulation frequency *f*_*m*_.

**Figure 4:**
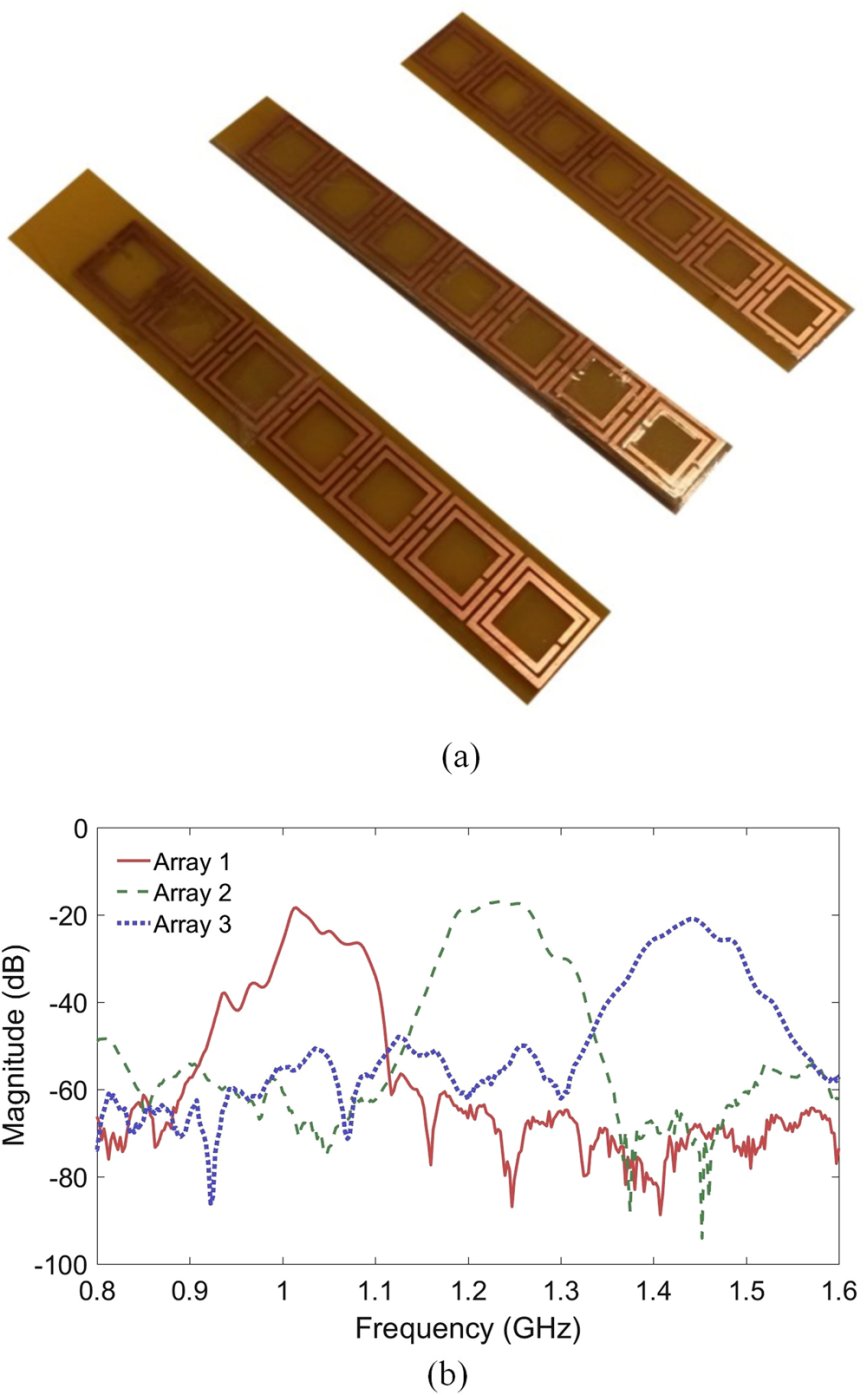
(**a**) Photography of the three metamaterial based waveguides made by SRR arrays. (**b**) Measured coefficient transmission of the designed arrays.

Figure 4(a) shows the three realized meta-waveguides. The three arrays of SRRs designed previously have been printed on an Epoxy substrate and disposed over a metallic ground plane. Two small antenna loops are used to characterize the waveguides. Figure 4(b) presents the measured spectra for the three meta-waveguides. One can note that the attenuation in the experiments is higher the one predicted by numerical simulations. This attenuation is due to higher actual losses and to the used cables. Nevertheless, a good agreement between the experimental and the numerical results is observed regarding the expected bandpass frequency domains.

**Figure 5:**
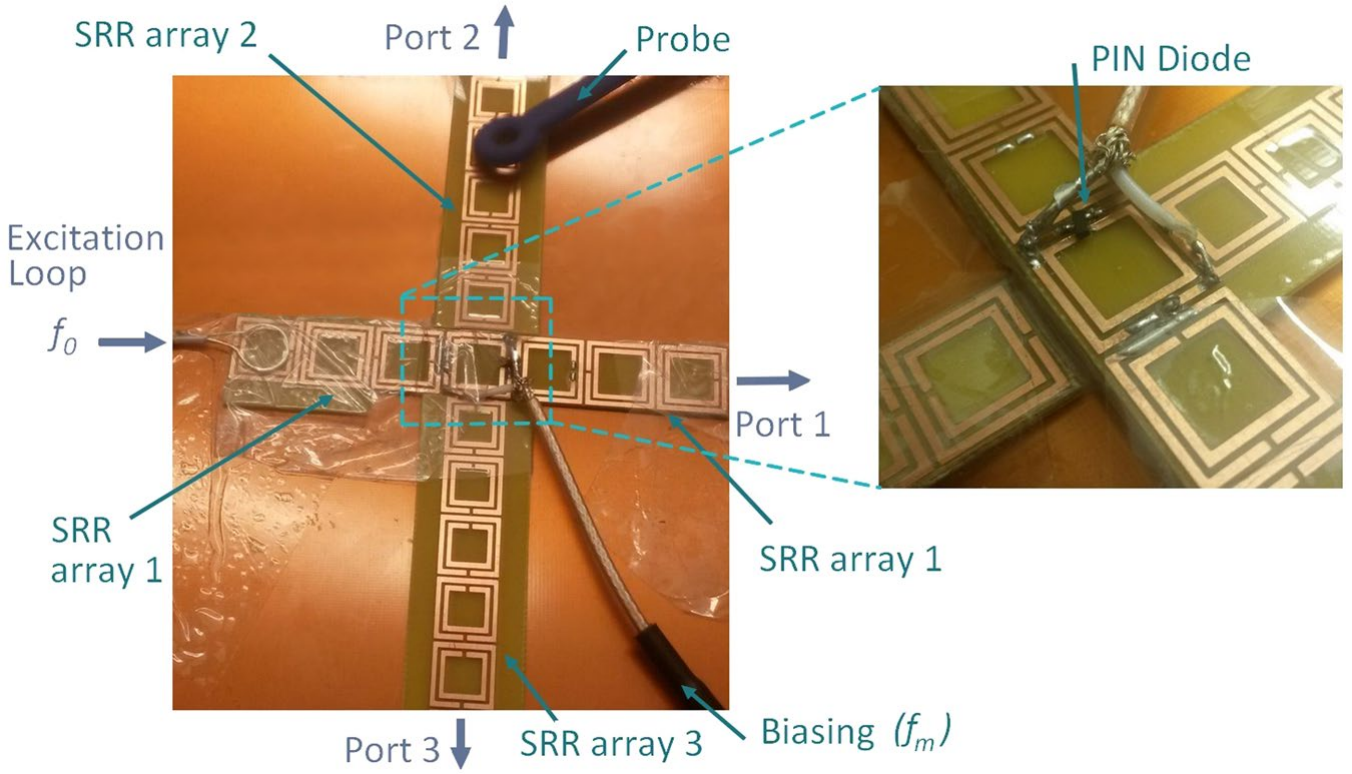
Photography of the metamaterial made by SRR arrays. A zoom on the time varying plasmonic cell is given on the right.

In order to validate experimentally the functioning of the time tarying and non-reciprocal switching device, the three waveguides are connected using an active metamaterial unit cell as illustrated in Fig. 5. The signal at the frequency *f*_0_ = 1 GHz is then injected in the main waveguide through a manufactured small loop antenna. Because this main waveguide supports SSP propagation at this frequency, the injected signal is able to reach the time-varying metamaterial junction. The modulation frequency *f*_*m*_ of the junction is controlled by the bias voltage signal provided by the SDR card. This signal, set to 10 dBm, is able to achieve the switching of the PIN diode. A small loop probe is used to measure the spectra at the output ports.

**Figure 6:**
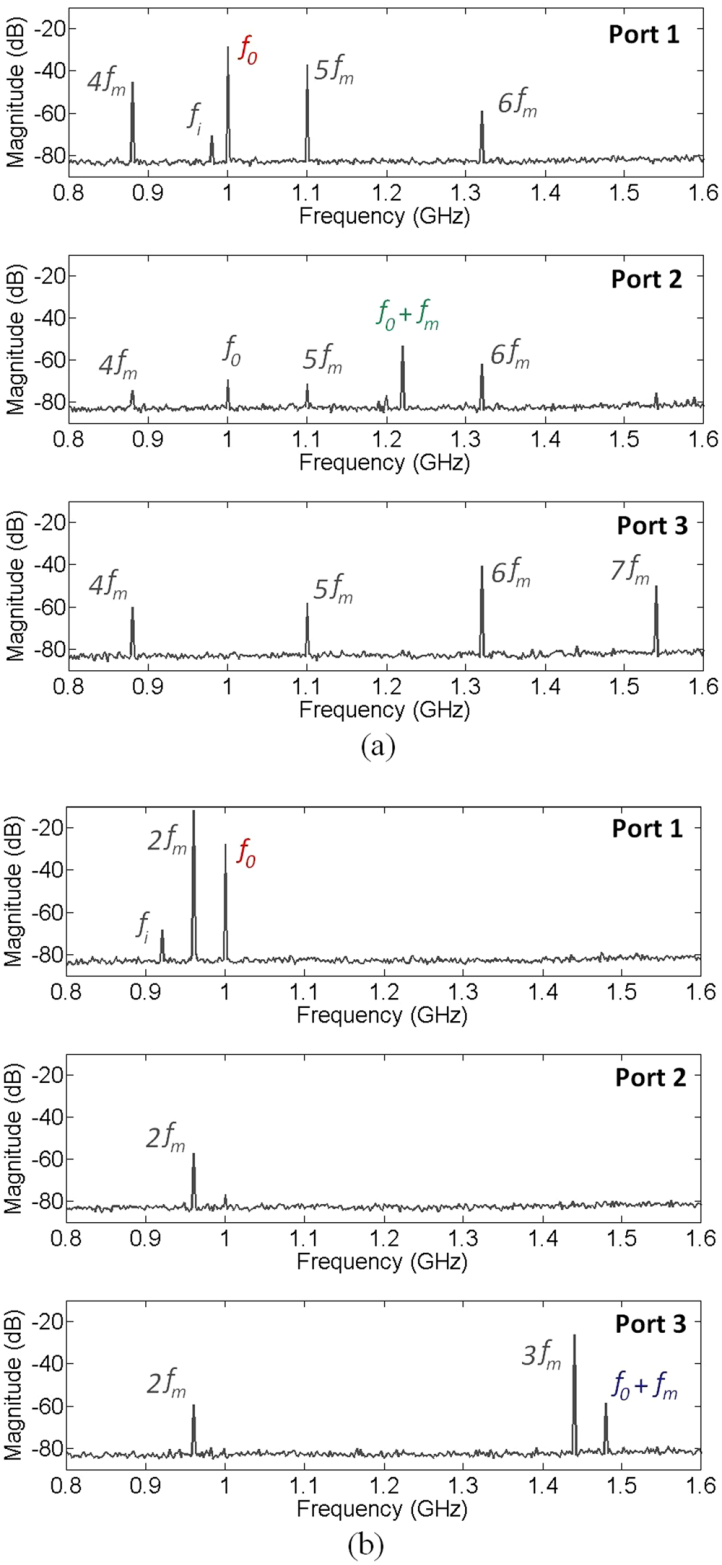
Measured spectra at the three ports for (**a**) the 220 MHz and (**b**) the 480 MHz modulation frequency.

Figure 6 shows the measured spectra at the three output ports for two different modulation frequency *f*_*m*_. In the first configuration, the modulation frequency is set to 220 MHz. In this case, the modulation of the injected signal in the junction leads to the generation of a supplementary signal at the frequency *f*_2_ = *f*_0_ + *f*_*m*_ = 1.22 GHz. At this frequency, the propagation of SP is possible only in the second waveguide. Because, SSP propagation at the frequency *f*_0_ is forbidden in this waveguide, only the signal at the frequency *f*_2_ is able to reach port 2. However, both signals, at *f*_0_ and at *f*_0_ + *f*_*m*_, should vanish at port 3. Despite a low level pic at the frequency *f*_0_ depiceted in the port 2 spectrum, this scenario is confirmed by the presented measurement of Fig. 6(a). The remaining signal at the frequency *f*_0_ is driven by the main waveguide to port 1 as illustrated by Fig. 6(a). Therefore, SP propagation is blueshifted to the frequency *f*_0_ + *f*_*m*_ and directed only to port 2 in this scenario.

One can notice here the existence of many pics not expected to appear in the measured spectra. These pics are essentially the harmonics of the modulation signal injected with a relatively high power. These harmonics are due to the non linear behavior of the PIN diode. We can also note here the appearance of one of the image frequencies at the frequency *f*_*i*_ = 0.98 GHz.

Figure 6(b) shows the measured spectra in the second configuration where the modulation frequency is *f*_*m*_ = 480 MHz. In this case, the frequency of the generated signal at the junction is *f*_3_ = *f*_0_ + *f*_*m*_ = 1.48 GHz. The propagation of SP at this frequency is possible only in the third waveguide. Thereby, this signal is observed only at port 3 as illustrated by Fig. 6(b). The remaining signal at the frequency *f*_0_ is driven to port 1 as in the first configuration.

Because the selective bandpasses of the second and the third waveguides, transmitting SPs from port 2 or port 3 at the frequency *f*_0_ is not possible. This phenomenon relates the non-reciprocal property of the proposed structure. However, sending back the shifted freuquency (*f*_2_ or *f*_3_) remains possible by considering the corresponding modulation frequency in each configuration.

With these two scenarios, the proposed structure is able to achieve a non reciprocal active switching of the injected signal by controlling the modulation frequency. This functioning is realized on a subwavelength device thanks to the small effective wavelengths provided by the SP dispersion relation. The obtained results demonstrate the possibility of controlling the propagation of SP by using time-varying metamaterials.

## Conclusion

In summary, we have proposed a time varying and non-reciprocal metamaterial to control the spoof plasmon propagation. A subwavelength device based on split ring resonators has been designed as an application of this concept. This non-reciprocal device is able to blue-shift the propagating SP waves and to provide an active steering of these SPs by controlling the modulation frequency the time varying metamaterial. By using such device, an active control of spoof plasmon propagation have been demonstrated experimentally in the microwave regime. Controlling the SP propagation as demonstrated could be realized by using different techniques that are able to impose an active variation of the effective parameters of metamaterials. This approach could be extended to infrared and optical regimes by considering suitable metamaterials and by employing specific techniques like the electro-optical modulation.

## Methods

### Numerical simulations

Full wave commercial software (CST Studio) has been used to perform the numerical characterization. The calculations has been accomplished by the time domain solver of this software. Typical parameters of the Epoxy and the copper materials have been specified in the presented simulation results. The magnetic field cartographies have been calculated by the solver and post-processed in the final display.

### Measurements

The experimental characterization of the structures has been performed in a semi-anechoic chamber. An Agilent spectrum analyzer has been employed to measure the transmission spectra. The USRP B210 software-defined radio (SDR) card is used to provide the bias voltage operating at the modulation frequency *f*_*m*_. The SDR has been controlled by a GNURADIO code where the maximum power has been set to 10 dBm. A manufactured small loop has been realized and used to excite the main waveguide. The measured signals have been collected using a commercial RF loop probe from AARONIA AG that was connected to the spectrum analyzer.

## References

[1] Raether, H. *Surface Plasmons* (Springer-Verlag, Berlin, 1988).

[2] Barnes WL, Dereux A, Ebbesen TW (2003). Surface plasmon subwavelength optics. Nature.

[3] Yin L (2005). Subwavelength focusing and guiding of surface plasmons. Nano Letters.

[4] Chen W, Abeysinghe DC, Nelson RL, Zhan Q (2009). Plasmonic lens made of multiple concentric metallic rings under radially polarized illumination. Nano Letters.

[5] Wang G, Lu H, Liu X, Mao D, Duan L (2011). Tunable multi-channel wavelength demultiplexer based on mim plasmonic nanodisk resonators at telecommunication regime. Opt. Express.

[6] Charbonneau R, Lahoud N, Mattiussi G, Berini P (2005). Demonstration of integrated optics elements based on long-ranging surface plasmon polaritons. Opt. Express.

[7] Ourir A, Fink M (2014). Subwavelength far-field imaging at visible and ultraviolet wavelengths using broadband surface plasmon waves. Phys. Rev. B.

[8] Pendry JB, Martin-Moreno L, Garcia-Vidal FJ (2004). Mimicking surface plasmons with structured surfaces. Science.

[9] Garcia-Vidal FJ, Martin-Moreno L, Pendry JB (2005). Surfaces with holes in them: new plasmonic metamaterials. Journal of Optics A: Pure and Applied Optics.

[10] Maier SA, Andrews SR, Martin-Moreno L, Garcia-Vidal FJ (2006). Terahertz surface plasmon-polariton propagation and focusing on periodically corrugated metal wires. Phys. Rev. Lett..

[11] Zhu W, Agrawal A, Nahata A (2008). Planar plasmonic terahertz guided-wave devices. Opt. Express.

[12] Fernandez-Dominguez AI, Moreno E, Martin-Moreno L, Garcia-Vidal FJ (2009). Guiding terahertz waves along subwavelength channels. Phys. Rev. B.

[13] Martin-Cano D (2010). Domino plasmons for subwavelengthterahertz circuitry. Opt. Express.

[14] Zhao W, Eldaiki OM, Yang R, Lu Z (2010). Deep subwavelength waveguiding and focusing based on designer surface plasmons. Opt. Express.

[15] Kim S-H (2011). Experimental demonstration of self-collimation of spoof surface plasmons. Phys. Rev. B.

[16] Kim K-J (2014). Propagation of spoof surface plasmon on metallic square lattice: bending and splitting of self-collimated beams. Opt. Express.

[17] Zhang HC, Fan Y, Guo J, Fu X, Cui TJ (2016). Second-harmonic generation of spoof surface plasmon polaritons using nonlinear plasmonic metamaterials. ACS Photonics.

[18] Shaltout A, Kildishev A, Shalaev V (2015). Time-varying metasurfaces and lorentz non-reciprocity. Opt. Mater. Express.

[19] Ourir A, Maurel A, Félix S, Mercier J-F, Fink M (2017). Manipulating light at subwavelength scale by exploiting defect-guided spoof plasmon modes. Phys. Rev. B.

[20] Vasudev AP, Kang J-H, Park J, Liu X, Brongersma ML (2013). Electro-optical modulation of a silicon waveguide with an “epsilon-near-zero” material. Opt. Express.

[21] Krasavin AV, Zayats AV (2012). Photonic signal processing on electronic scales: Electro-optical field-effect nanoplasmonic modulator. Phys. Rev. Lett..

[22] Liu L, Yang C, Yang J, Xiang H, Han D (2017). Spoof surface plasmon polaritons on ultrathin metal strips: from rectangular grooves to split-ring structures. J. Opt. Soc. Am. B.

[23] Guth N (2012). Optical properties of metamaterials: Influence of electric multipoles, magnetoelectric coupling, and spatial dispersion. Phys. Rev. B.

[24] Syms RRA, Shamonina E, Solymar L (2006). Magneto-inductive waveguide devices. IEE Proceedings - Microwaves, Antennas and Propagation.

